# Integrating TCGA and Single-Cell Sequencing Data for Hepatocellular Carcinoma: A Novel Glycosylation (GLY)/Tumor Microenvironment (TME) Classifier to Predict Prognosis and Immunotherapy Response

**DOI:** 10.3390/metabo14010051

**Published:** 2024-01-13

**Authors:** Yun Wu, Jiaru Chen, Riting Zhu, Guoliang Huang, Jincheng Zeng, Hongbing Yu, Zhiwei He, Cuifang Han

**Affiliations:** 1 Guangdong Provincial Key Laboratory of Medical Molecular Diagnostics, Guangdong Medical University, Dongguan 523808, China; wuyun@gdmu.edu.cn (Y.W.); cjrkkl@gdmu.edu.cn (J.C.); zhuriting1998@gdmu.edu.cn (R.Z.); huangguoliang@gdmu.edu.cn (G.H.); zengjc@gdmu.edu.cn (J.Z.); hbyu@gdmu.edu.cn (H.Y.); 2 School of Pharmacy, Guangdong Medical University, Dongguan 523808, China; 3 Dongguan Key Laboratory of Medical Bioactive Molecular Developmental and Translational Research, Guangdong Medical University, Dongguan 523808, China

**Keywords:** glycosylation modification, tumor immune microenvironment, hepatocellular carcinoma, single-cell sequencing, malignant epithelial cells, prognosis

## Abstract

The major liver cancer subtype is hepatocellular carcinoma (HCC). Studies have indicated that a better prognosis is related to the presence of tumor-infiltrating lymphocytes (TILs) in HCC. However, the molecular pathways that drive immune cell variation in the tumor microenvironment (TME) remain poorly understood. Glycosylation (GLY)-related genes have a vital function in the pathogenesis of numerous tumors, including HCC. This study aimed to develop a GLY/TME classifier based on glycosylation-related gene scores and tumor microenvironment scores to provide a novel prognostic model to improve the prediction of clinical outcomes. The reliability of the signatures was assessed using receiver operating characteristic (ROC) and survival analyses and was verified with external datasets. Furthermore, the correlation between glycosylation-related genes and other cells in the immune environment, the immune signature of the GLY/TME classifier, and the efficacy of immunotherapy were also investigated. The GLY score low/TME score high subgroup showed a favorable prognosis and therapeutic response based on significant differences in immune-related molecules and cancer cell signaling mechanisms. We evaluated the prognostic role of the GLY/TME classifier that demonstrated overall prognostic significance for prognosis and therapeutic response before treatment, which may provide new options for creating the best possible therapeutic approaches for patients.

## 1. Introduction

HCC is a malignant liver tumor with a global incidence and has a complex etiology, an insidious onset, and an elevated malignancy degree [1]. Currently, targeted chemotherapy and immunotherapy are still effective treatments for advanced HCC [2,3,4]. Multikinase inhibitors inhibit tumor proliferation, angiogenesis, metastasis, and invasion by targeting multiple intracellular and cell surface kinases, including sorafenib, lenvatinib, regorafenib, cabozantinib, and ramucirumab, which have been approved as targeted treatments for HCC [5]. To date, immunotherapy, particularly immune checkpoint inhibitors (ICIs), has shown efficacy in HCC, but the complex tumor immune microenvironment (TIME) of HCC has somewhat limited the clinical response rate to current mainstream drug therapy and T-cell-based immunotherapy [6,7]. Crosstalk between tumor cells and the surrounding TME has been implicated in tumorigenesis and immune evasion [8,9]. Comprehensive mining of the hidden information in the TME is crucial for gaining insight into HCC and improving treatment methods [10].

Glycosylation is the most abundant and diverse form of post-translational modification of proteins that is common to all eukaryotic cells [11]. Advancements in cancer-associated glycoproteomics have provided new insights into the mechanisms driving tumor initiation and progression [12]. Glycosylation abnormalities are frequently observed in HCC and contribute to changes in protein properties and functions. Altered protein glycosylation plays a critical role in carcinogenesis, tumor progression, metastasis, stemness, recurrence, immune evasion, and therapy resistance, underscoring the significance of glycosylation abnormalities as potential diagnostic and prognostic biomarkers in HCC [13].

Currently, immunotherapy is a promising therapeutic strategy for HCC [7]. Immune checkpoint inhibitors, oncolytic viroimmunotherapy, and adoptive T-cell transfer have been developed for the immunotherapy of HCC. However, as a new mainstream therapy, immunotherapy for HCC still has unresolved challenges, including the role of immunotherapy in the early phases of HCC, evaluating the use of combined strategies targeting the immune microenvironment and checkpoint inhibition, addressing the immunosuppressive components of HCC in the tumor immune microenvironment, and developing therapeutic approaches for patients who do not respond to current immunotherapy [14]. In view of the above issues and challenges, it is very important to find reliable molecular biomarkers. In this investigation, we examined the ability of the GLY/TME classifier, and the results indicated that the GLY^low^/TME^high^ subgroup had the highest number of patients who responded to immunotherapy, suggesting that this classifier could predict immunotherapy response and better survival. In addition, the proteomic patterns of the GLY^low^/TME^high^ group and the HCC immunotherapy responder group showed extremely high similarity, which may reveal certain commonalities in the decisive interaction between the tumor cells and immune system. This demonstrated the treatment predictive power of the GLY/TME classifier.

## 2. Materials and Methods

### 2.1. Data Collection and Preparation

University of California, Santa Cruz (UCSC) Xena (https://xena.ucsc.edu/) stores collated clinical data from the The Cancer Genome Atlas (TCGA) database for various cancer types, and we downloaded genomic data and clinical annotations of Liver Hepatocellular Carcinoma (LIHC) patients from the UCSC Xena database and Gene Expression Omnibus (GEO, https://www.ncbi.nlm.nih.gov/geo/, accessed on 11 June 2023) database, which included LIHC (*n* = 374) and normal (*n* = 50) samples on 11 June 2023, and then performed log2 TPM (transcripts per kilobase million) +1 formats for each expression value. In total, 770 glycosylation-related genes (Supplementary Table S1) were collected from the Molecular Signatures Database (MSigDB version 7.2, https://www.gseamsigdb.org/gsea/msigdb, accessed on 12 June 2023).

Single-cell transcriptome data for 18,985 cells (2 HCC samples) were downloaded from the GEO database (data set GSE166635) and normalized the expression matrix of the top 2000 highly variable genes by the “Seurat” package [15]. Batch effects were minimized utilizing the “RunHarmony” function of the “harmony” package [16]. Cell annotation was identified manually based on canonical marker genes from the CellMarker 2.0 (http://bio-bigdata.hrbmu.edu.cn/CellMarker, accessed on 20 June 2023), and t-distributed stochastic neighbor embedding (t-SNE) was used to show cell distributions [17].

### 2.2. CIBERSORT Analysis

We analyzed the impact of 22 immune cell types on HCC prognosis using Cell-type Identification By Estimating Relative Subsets Of RNA Transcripts (CIBERSORT) based on RNA-seq data, and Kaplan–Meier (KM) survival analysis was utilized to examine the differential abundance of immune cells in overall survival (OS) in HCC [18].

### 2.3. Formation and Verification of the GLY/TME Scoring System

The “limma” R package (version 3.50.3) was utilized to find differentially expressed genes (DEGs) across cancerous and paracancerous tissues [19], and then, overlapping DEGs were identified by intersecting the screened DEGs with the collected genes related to glycosylation modifications. Univariate Cox regression analysis was conducted on the assembled training cohort to identify overlapping DEGs by using the “survival” R package (version 3.3-1), and 15 significant predictors were obtained by employing least absolute shrinkage and selection operator (LASSO) regression analysis. Here, the bootstrap method was used to resample the TCGA-LIHC samples 1000 times to obtain a stable signature. Finally, multivariate Cox regression analysis was used to construct a glycosylation scoring system, and the risk score for glycosylation modifications in HCC was determined using the logistic regression formula as follows:

$$GLY\;score=\Sigma \frac{Coefi*Gi}{Bootstrap(SD)}$$

where i indicates the number of hub genes and Coef i is each gene’s coefficient obtained by multivariate Cox regression analysis. Gi is each gene’s expression level and bootstrap (SD).

Establishing the TME score depended on CIBERSORT analysis, and the scores were selected by using KM survival analysis:

$$TME\;score=\Sigma \frac{-Coefi*Cj}{Bootstrap(SD)}$$

where Cj is the abundance of prognosis-related immune cells.

Cutoff values for high and low grouping were detected by utilizing the “survminer” R package (version 0.4.9). Then, KM curves were obtained by applying the log-rank test to the training and validation datasets. Finally, a combined prognostic analysis of the GLY score and TME score was performed to obtain a GLY/TME classifier which comprised four groups in the TCGA-LIHC cohort: GLY^high^/TME^low^, GLY^low^/TME^high^, GLY^high^/TME^high^, and GLY^low^/TME^low^.

### 2.4. Weighted Gene Coexpression Network Analysis (WGCNA)

Utilizing the “WGCNA” program, characteristic genes of the four groups mentioned above were determined [20]. A gene coexpression network based on the top 5000 genes was created after aberrant samples were eliminated, and it was further converted into a scale-free network by choosing the proper soft-threshold power β. The eigengene adjacency and the topological overlap measure (TOM) were generated to assess the interactions between gene modules, and the greatest correlation was chosen for further study based on the association between the eigenvalue of the module and the phenotype.

### 2.5. Functional Enrichment Analysis

The “clusterProfiler” R package (v4.2.2) [21] and fast gene set enrichment analysis (FGSEA) (v1.20.0) were used to perform gene set enrichment analysis (GSEA) of the biological processes in the Kyoto Encyclopedia of Genes and Genomes (KEGG) and MSigDB database, respectively. Notably, |NES| > 1 and padj < 0.05 were set as the threshold for differential gene screening. The top 20 pathways enriched for each group were displayed by utilizing the “pheatmap” (version 1.0.12) R package [22].

To understand the biological significance of the signature genes identified by the WGCNA, the genes of hub module were mapped into Metascape for KEGG pathway enrichment analysis and gene ontology enrichment analysis.

We then visualized the abundance and role of proteins, and the KEGG database platform was used to annotate these proteomaps [23]. Here, we visualized the proteomaps of GLY^low^/TME^high^ patients receiving immunotherapy and those responding to immunotherapy.

### 2.6. TIP Analysis

In bulk tumor samples, a GSEA-based technique is used to evaluate the relative immunological activity of the immune cycle [24], and a variety of tumor-infiltrating immune cells currently tracking the tumor immunophenotype (TIP) are derived from CIBERSORT [25].

### 2.7. Immunotherapy Response Prediction

Tumor Immune Dysfunction and Exclusion (TIDE), an online algorithm that uses transcriptome information to anticipate patients’ clinical response to immune checkpoint blockade treatments, was employed to anticipate the treatment sensitivity of patients associated with glycosylation and high and low scores for the immunological microenvironment [26].

### 2.8. Intercellular Communications

The potential interactions between malignant cells with different GLY score and other cells were employed by the “CellChat” (1.5.0) package [27].

### 2.9. Cell Culture and mRNA and Protein Level Analysis

The normal human hepatocyte cell lines MIHA and HCC-LM3 were cultured in RPMI-1640 and DMEM (Gibco, Waltham, MA, USA) with 10% FBS (Sigma-Aldrich, St. Louis, MO, USA) in a 5% CO_2_ environment at 37 °C. Total RNA was extracted, and an Omniscript Reverse Transcriptase kit (Qiagen, Hilden, Germany ) was applied to reverse-transcribe 2 μg of total RNA. mRNA level of the target and normalization to that of beta-actin mRNA level were detected by quantitative real-time PCR (qRT-PCR) using the 2−ΔΔCt method and the primers are listed in Supplementary Table S2. The primary antibodies used in this study were PPIA Ab (No.ab126738, Abcam, Cambridge, UK), ALG3 Ab (No.20290-1-AP, Proteintech, Rosemont, IL, USA), CTSA Ab (No.ab184553, Abcam), CAD Ab (No. ab40800, Abcam), B3GAT3 Ab (No.SAB1401628, Sigma-Aldrich), TRAPPC3 Ab (No.15555-1-AP, Proteintech), HSP90AA1 Ab (No.GTX109753, GeneTex, CA, USA), SRD5A3 Ab (No.NBP1-69612, Novus Biologicals, Littleton, CO, USA), BAG2 Ab (No.ab79406, Abcam), DNAJC1 Ab (No.GTX103858, GeneTex), ADAMTS5 Ab (No. ab182795, Abcam), PLOD2 Ab (No. DF12018, Affinity Biosciences, Cincinnati, OH, USA), DYNC1LI1 Ab (No.GTX120114, GeneTex), ST6GALNAC4 Ab (No. ab127016, Abcam), calcium-binding protein p22 (CHP1) Ab (No. GTX113936, GeneTex), and β-actin (No. T0022, Affinity Biosciences). The Clinical Proteomic Tumor Analysis Consortium (CPTAC) was used to evaluate the protein levels in HCC patients [28].

### 2.10. Prediction of Drug Response

The “oncoPredict” R package was employed to explore the differences in drug treatment effects among HCC patients [29]. The data of cell lines to drugs in the Genomics of Drug Sensitivity in Cancer Project (GDSC) were used as the input data. A lower half-maximal inhibitory concentration (IC50) value indicates a higher drug efficacy.

### 2.11. Statistical Analysis

The R (v4.1.2) program was employed to conduct all statistical analyses and the Wilcoxon rank-sum test was utilized to assess the differences between the two groups. Variations between three or more groups were evaluated by using the Kruskal–Wallis test. Spearman’s correlation coefficient was employed to evaluate correlations. The standard for statistical significance was *p* < 0.05.

## 3. Results

### 3.1. Construction of GLY Score and TME Score

First, based on the MSigDB database, we collected a gene set containing 770 glycosylation-related genes by searching for the keyword “glycosylation” after removing duplicate genes. The differentially expressed glycosylation-related genes between tumors and nearby healthy tissues were determined by utilizing “limma” package, (Supplementary Table S1). Typically, 603 differentially expressed glycosylation-related genes were recognized from 770 glycosylation-related genes by setting the screening threshold as *p* value = 0.05 after removing genes that were not expressed across most samples. Then, 301 DEGs associated with prognosis were screened out by univariate Cox analysis, and the differential expression of these glycosylation-related genes in HCC compared to normal paracancerous tissues was shown using a heatmap (Figure S1A, Supplementary Table S3).

**Figure 1:**
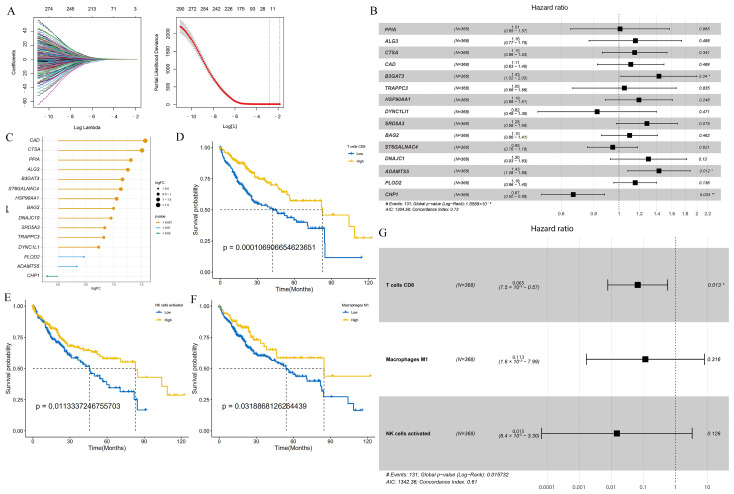
The GLY score and the TME score were developed in the training cohort based on glycosylation-related genes and tumor immune microenvironment cells. (**A**) Left panel: tenfold cross-validation of variable selection in LASSO regression using the minimum criteria. Right panel: LASSO coefficients for glycosylation. Each curve represents one glycosylation-related gene. (**B**) Forest plot showing gene expression and the OS outcomes. (**C**) The lollipop plot represents the logFC values of 15 glycosylation-related prognostic genes. A logFC > 0 indicates upregulated genes; logFC < 0 indicates downregulated genes. The colors represent the different significance levels of the gene, where orange represents *p* value < 0.001, blue represents *p* value < 0.01, and green represents *p* value < 0.05. (**D**–**F**) KM curves indicate that patients with high infiltration of activated NK cells, M1 macrophages, and CD8 T cells have a better prognosis. (**G**) Forest plot revealing the predictive value of three immune cells correlated with a good prognosis in HCC (HR < 1). (* *p* < 0.05, ** *p* < 0.01).

Next, LASSO regression analysis was utilized to select glycosylation-related genes with non-zero penalty coefficients, which generated a prognosis-related glycosylation gene signature (Figure 1A). Finally, a robust prognostic gene signature was constructed with 15 genes, namely, *PPIA*, *ALG3*, *CTSA*, *CAD*, *B3GAT3*, *TRAPPC3*, *HSP90AA1*, *SRD5A3*, *BAG2*, *DNAJC1*, *ADAMTS5*, *PLOD2*, *DYNC1LI1*, *ST6GALNAC4*, *CHP1*, and detailed information about them is provided in Supplementary Table S4. The forest plot showed that the hazard ratios (HRs) of *PPIA*, *ALG3*, *CTSA*, *CAD*, *B3GAT3*, *TRAPPC3*, *HSP90AA1*, *SRD5A3*, *BAG2*, *DNAJC1*, *ADAMTS5*, and *PLOD2* were all greater than 1, indicating that these 12 genes may be risk prognostic factors. DYNC1LI1, ST6GALNAC4, and CHP1 were less than 1, which may be favorable prognostic factors (Figure 1B). The lollipop plot shows the gene expression fold change (logFC) of glycosylation-related prognostic genes in the TCGA-LIHC cohort, where a positive logFC indicates upregulation of gene expression in HCC compared to normal tissue, while a negative logFC indicates downregulation of genes (Figure 1C).

Next, we constructed the relative scores of immune cells in the HCC samples and combined the survival curve of each cell with the optimal cutoff to screen for immune cells associated with good prognosis, such as natural killer cells (NK cells), M1 macrophages, and activated CD8 T cells (Figure 1D–F and Figure S1B). Forest plots showed that the HR values of these three cell types were all less than 1, suggesting that these cell types were protective factors (Figure 1G). Bootstrap resampling and multivariate Cox regression analysis were further performed to construct a TME score.

### 3.2. Differential mRNA and Protein Levels of Glycosylation-Related Prognostic Genes

**Figure 2:**
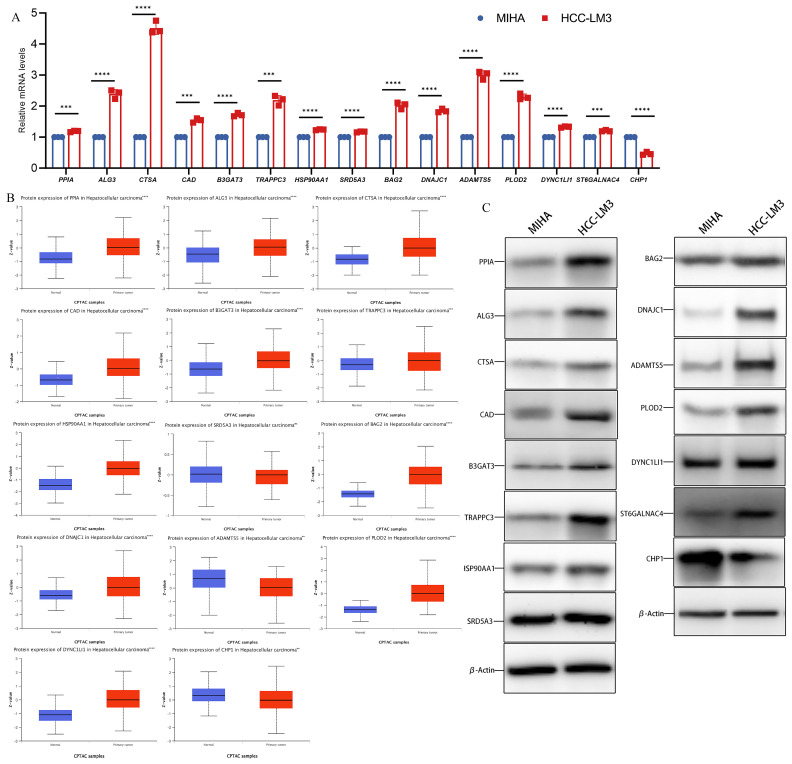
The relative expression level of glycosylation-related prognostic genes. (**A**) The relative mRNA level of *PPIA*, *ALG3*, *CTSA*, *CAD*, *B3GAT3*, *TRAPPC3*, *HSP90AA1*, *SRD5A3*, *BAG2*, *DNAJC1*, *ADAMTS5*, *PLOD2*, *DYNC1LI1*, *ST6GALNAC4*, and *CHP1*. (**B**) Expression of 15 hub genes in HCC primary tumor samples and adjacent normal tissues at the protein level from the CPTAC database (*n* = 165). (**C**) Expression of 15 hub genes in MIHA cell and HCC-LM3 cell at the protein level. (Data are presented as the mean ± SD, ** *p* < 0.01, *** *p* < 0.001, **** *p* < 0.0001, and ns, no significance.).

The mRNA levels of 15 genes were compared by qPCR in normal human hepatocytes MIHA and HCC-LM3 with high metastatic potential. The results revealed that CHP1 had low expression and *PPIA*, *ALG3*, *CTSA*, *CAD*, *B3GAT3*, *TRAPPC3*, *HSP90AA1*, *SRD5A3*, *BAG2*, *DNAJC1*, *ADAMTS5*, *PLOD2*, *DYNC1LI1*, and *ST6GALNAC4* were significantly overexpressed in human hepatocellular carcinoma cells compared to MIHA (Figure 2A). Moreover, according to the CPTAC database, the protein level expression of *CHP1* was lower and *PPIA*, *ALG3*, *CTSA*, *CAD*, *B3GAT3*, *TRAPPC3*, *HSP90AA1*, *BAG2*, *DNAJC1*, *PLOD2*, *DYNC1LI1*, and *ST6GALNAC4* were significantly higher in tumor tissues. The protein expression of *SRD5A3* was not significantly different between tumor and normal tissues (Figure 2B). We detected the expression of 15 hub gene expressed proteins in MIHA cells and HCC-LM3 cells for comparison through western blot analysis. The results revealed that *CHP1* had low expression and *PPIA*, *ALG3*, *CTSA*, *CAD*, *B3GAT3*, *TRAPPC3*, *HSP90AA1*, *SRD5A3*, *BAG2*, *DNAJC1*, *ADAMTS5*, *PLOD2*, *DYNC1LI1*, and *ST6GALNAC4* were overexpressed in HCC-LM3 cells compared to MIHA (Figure 2C).

### 3.3. GLY/TME Score Performs Well in Predicting Prognosis

**Figure 3:**
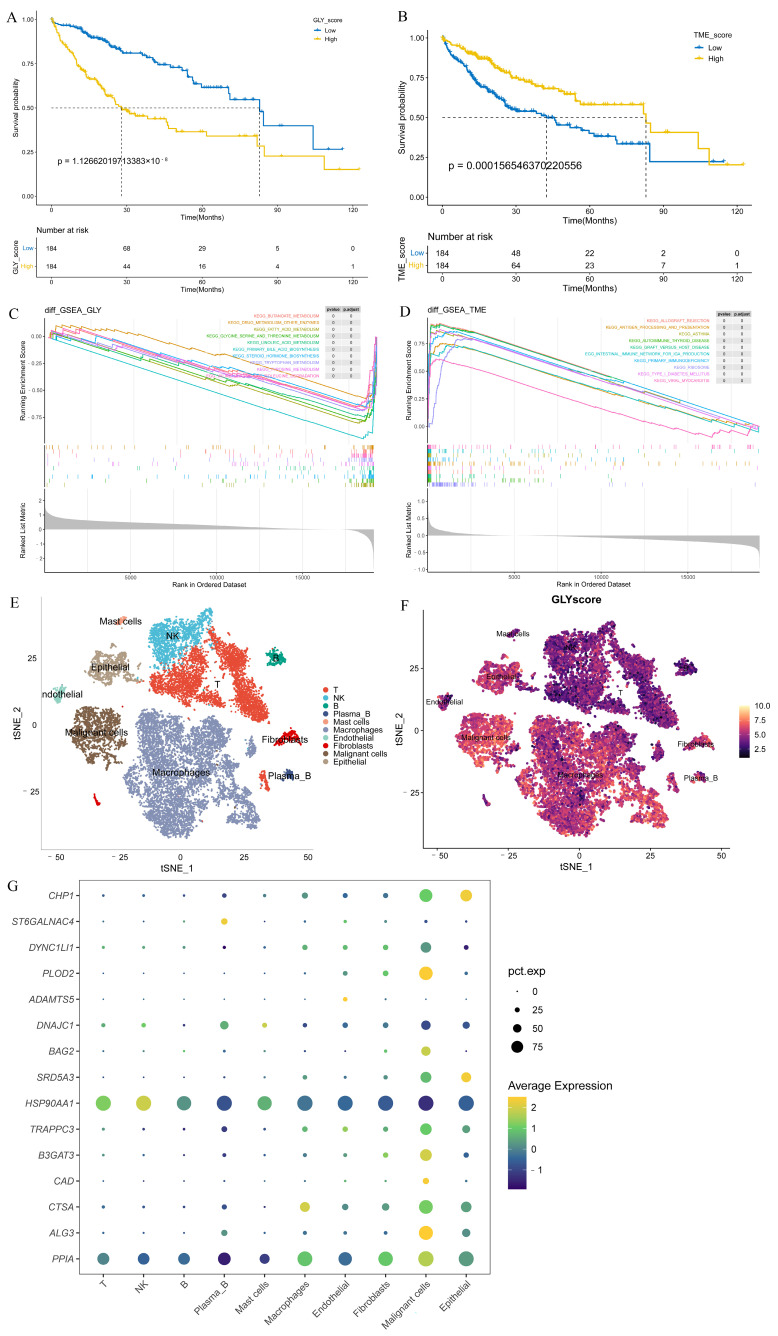
Evaluation of the prognostic power of GLY score and TME score. (**A**) KM survival analysis of the GLY score groups. The yellow and blue lines represent the high and low GLY scores, respectively. (**B**) The KM survival curve shows that patients with a low TME score in the training dataset had significantly shorter OS than those with a high TME score (*p* < 0.001). (**C**) GSEA demonstrated that the hallmark significantly enriched pathways. The high GLY score is located on the left of the origin of the *x*-axis, and the low GLY score is located on the right of the *x*-axis. (**D**) The hallmark pathways were significantly enriched in both the low- and high-TME score groups. The high TME score is to the left of the origin of the *x*-axis, whereas the low TME score is to the right. (**E**) The t-SNE plot of all 18,985 cells collected from 2 individuals with primary liver cancer. Each color represents one cell type. (**F**) t-SNE plots colored according to GLY score. (**G**) Differential expression of 15 glycosylation-related prognostic genes in all cell types.

Patients in the training cohort were distributed into high- and low-GLY groups and TME groups based on the corresponding median value to evaluate the prognostic power of GLY score and TME score for HCC. Patients with a high GLY score had a significantly worse OS compared to those with a low GLY score (*p* < 0.001) (Figure 3A). Conversely, patients with a high TME score had longer survival (Figure 3B). Pathway enrichment analyses were conducted on the high- and low-GLY score and TME score groups. The GSEA results showed that the high- and low-GLY score groups were primarily related to fatty acid metabolism, tryptophan metabolism, butyrate metabolism, and other metabolic pathways (Figure 3C). Immune-related pathways, such as antigen processing and presentation and primary immunodeficiency, are commonly enriched pathways in both high- and low-TME score groups (Figure 3D).

Notably, we used the t-SNE method on single-cell data from HCC biospecimens from two individuals, and the GLY score was generated for each cell subset to further verify the glycosylation-related gene scores in the HCC single-cell transcriptomic landscape. Using known marker genes and published annotations from the literature, we identified epithelial cells (*CD24*, *KRT19*, *EPCAM*), malignant cells (*TTR*, *AMBP*, *APOH*), fibroblasts (*ACTA2*, *RGS5*, *TAGLN*), endothelial cells (*PECAM1*, *VWF*, *CDH5*), macrophages (*CSF1R*, *CPVL*, *CD68*), mast cells (*GATA2*, *KIT*, *CPA3*), plasma B cells (*MZB1*, *JCHAIN*, *IGHG1*), B cells (*MS4A1*, *BANK1*, *TNFRSF13C*), NK cells (*NKG7*, *GZMK*, *PRF1*), and T cells (*CD3G*, *CD2*, *CD3D*) (Figure 3E). We found higher GLY scores in malignant cells and epithelial cells than in immune cells (Figure 3F). Next, we sought to confirm in which cell types the 15 prognosis-related genes were highly expressed. We discovered that all of these prognosis-related genes were mainly expressed in tumor-related cells (such as malignant and epithelial cells) at the single-cell level, but their expression levels in immune cells were very low (Figure 3G).

### 3.4. Construction and Prognostic Value Assessment of the GLY/TME Classifier

**Figure 4:**
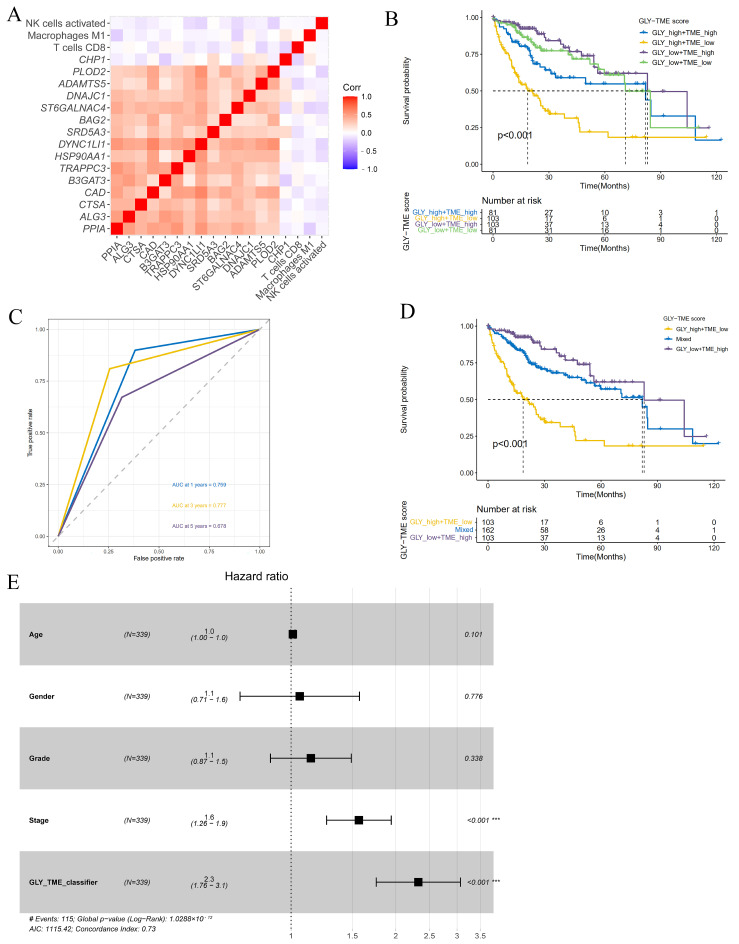
Construction and evaluation of the GLY/TME classifier. (**A**) Heatmap showing the correlations between 15 glycosylation-related prognostic genes and three prognostic-related immune cells. (**B**) Utilizing KM curves and the log-rank test, the prognoses of patients in the four subgroups were compared (GLY^low^/TME^high^, GLY^low^/TME^low^, GLY^high^/TME^high^, and GLY^high^/TME^low^). (**C**) The AUCs of ROC curves according to the GLY/TME classifier for predicting survival of HCC patients in the training cohort. (**D**) KM survival curves in the training cohort according to the GLY/TME classifier divided into three different subgroups (GLY^low^/TME^high^, mixed, and GLY^high^/TME^low^). Log-rank test, *p* < 0.001. (**E**) Multivariate Cox regression analysis of the GLY/TME classifier in the training cohort. (*** *p* < 0.001).

To better explore the association between immune-infiltrating cells and glycosylation-related prognostic genes in the TME, we analyzed the relationship between glycosylation-related prognostic genes and three immune-infiltrating cells. The correlation heatmap showed that, except for the *CHP1* gene, the remaining glycosylation-related prognostic genes were significantly positively associated with each other, while the three types of immune cells were negatively associated with these prognostic genes, indicating that the GLY score was generally associated with the TME (Figure 4A). Based on the significant correlation between cells with high immune infiltration and prognostic genes related to glycosylation modification, we combined the GLY score and the TME score for prognostic evaluation. The median values were used to divide the dataset into the GLY^high^/TME^low^, GLY^low^/TME^high^, GLY^high^/TME^high^, and GLY^low^/TME^low^ groups. Survival analysis of the four subgroups further demonstrated that the GLY^high^/TME^low^ subgroup had the worst prognosis, and the GLY^low^/TME^high^ subgroup had the best prognosis (Figure 4B).

In addition, to better understand whether the GLY/TME scoring system could effectively predict the prognosis of individuals with HCC, ROC curves of the two subgroups with the most obvious difference (GLY^high^/TME^low^ and GLY^low^/TME^high^) were plotted. The AUC values were 0.759, 0.777, and 0.678 at 1, 3, and 5 years, respectively, revealing that this scoring system was a good predictor of survival (Figure 4C). To further improve the GLY/TME scoring system, we combined the GLY^high^/TME^high^ and GLY^low^/TME^low^ subgroups with small prognostic differences into a mixed group, resulting in the GLY/TME classifier containing three types. Survival analysis showed a significant difference in prognostic status between the three types, with the GLY^low^/TME^high^ subgroup remaining the group with the longest OS (Figure 4D).

In the training cohort, the prognostic and predictive significance of the GLY/TME classifier was conducted by univariate and multivariate Cox regression analyses (Figure 4E and Figure S2A). And the results showed that the GLY/TME score was linked to OS in univariate Cox regression analysis and strongly linked to prognosis (*p* < 0.001), demonstrating that the GLY/TME scoring system was able to independently anticipate the clinical outcomes of individuals with HCC. Furthermore, the predictive value of the GLY/TME classifier was verified in the GSE14520 cohort (Figure S2B).

### 3.5. Different Underlying Molecular Mechanisms in the GLY/TME Classifier

**Figure 5:**
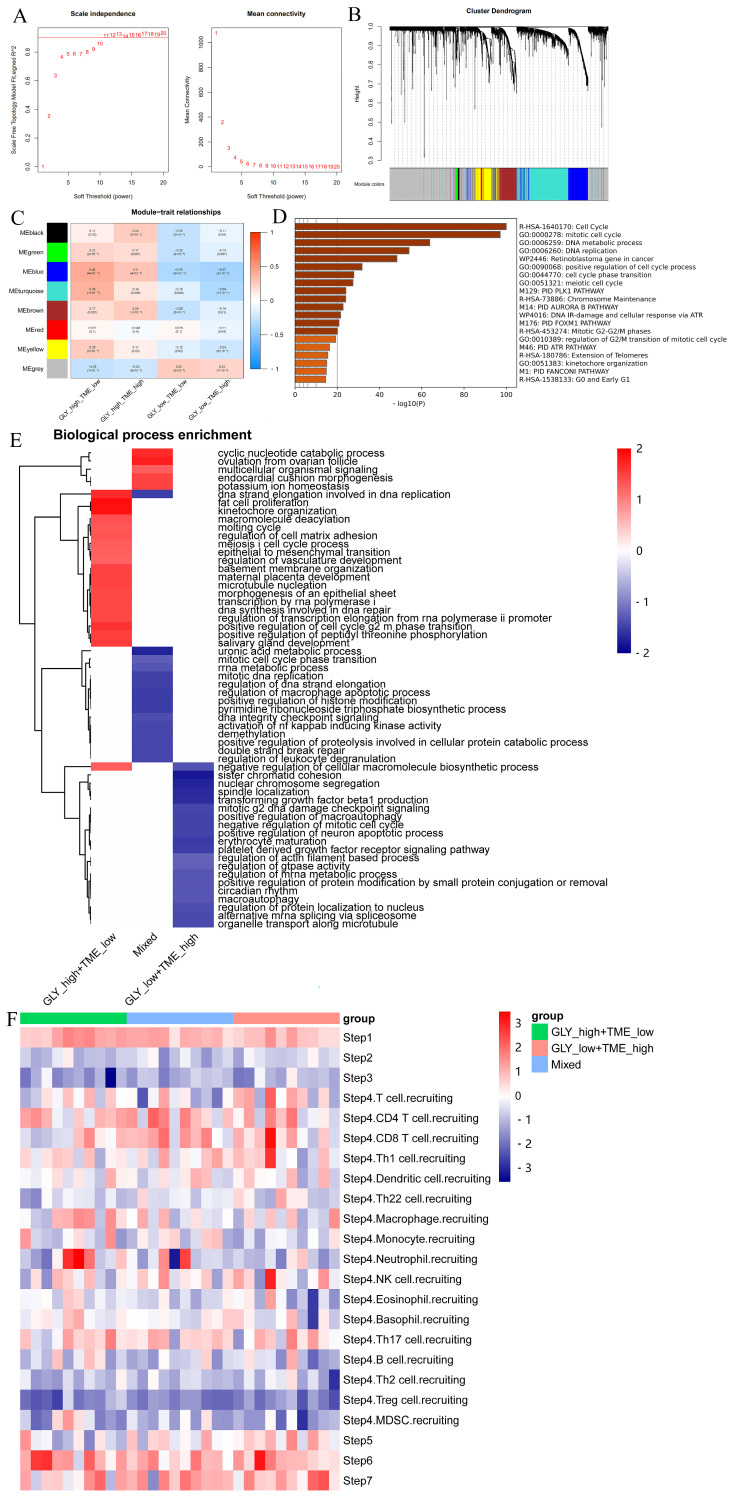
Identification of signature genes and particular biological mechanisms. (**A**) WGCNA was used to analyze the network topology for various soft-threshold powers. The right panel shows the effect of soft-threshold power on average connectivity, while the left panel shows the effect of soft-threshold power on the scale-free topology fit index. (**B**) Dendrogram (cluster tree) according to different metrics. (**C**) Heatmap of correlations between module signature genes and molecular phenotypes. (**D**) Pathway enrichment analysis of module genes in the GLY^High^/TME^low^ subgroup. (**E**) FGSEA showed the enriched BP pathways in the GLY^high^/TME^low^ subgroup, GLY^low^/TME^high^ subgroup, and mixed subgroup. (**F**) TIP database was used to visualize the variation in immune pathways across the three subgroups.

The “WGCNA” package was used to detect the signature genes of four subgroups. Outlier samples were first eliminated, and then the remaining samples were clustered. The scale-free R2 is 0.9 when β is set to 11, forming a scale-free network (Figure 5A). Next, eight coexpression modules were acquired via dynamic tree cutting and represented a clustering dendrogram (Figure 5B). A heatmap represents the eigengene adjacency of each module (Figure 5C). Furthermore, the correlation between the modules and the four cohorts was also measured. Among them, the blue module was significantly positively correlated with the GLY^high^/TME^low^ subgroup and negatively correlated with the GLY^low^/TME^high^ subgroup. Thus, we selected the signature genes of this module and performed enrichment analysis and showed that the genes in this module were primarily enriched in the cell cycle processes, indicating that the GLY^high^/TME^low^ subgroup was very strongly enriched in cell-cycle- and proliferation-related genes (Figure 5D).

To explore the biological features specific to the three subgroups, we performed GSEA on the DEGs utilizing the “fgsea” package (Figure 5E). The results showed that the GLY^high^/TME^low^ subgroup was mainly involved in the cyclic nucleotide catabolic process, DNA strand elongation involved in DNA replication, adipocyte proliferation, meiosis I cell cycle process, epithelial-to-mesenchymal transition, regulation of vasculature development, transcription by RNA polymerase I, DNA synthesis involved in DNA repair, regulation of transcriptional elongation from the RNA polymerase Ⅱ promoter, and positive regulation of the G2/M phase transition of the cell cycle. Simultaneously, in the GLY^low^/TME^high^ subgroup, the enriched pathways mainly included negative regulation of the cellular macromolecular biosynthesis process, mitotic G2 DNA damage checkpoint signaling, negative regulation of mitotic cell cycle, and other processes, which were antagonistic to the biological processes of the GLY^high^/TME^low^ subgroup.

To further explore the immune status of the three subgroups in the GLY/TME classifier, we therefore profiled the immune activity profiles of samples from patients with three types of HCC by using the TIP server algorithm. Finally, we visualized differences in immune-related pathways between the three subgroups (Figure 5F). Patients in the GLY^low^/TME^high^ subgroup had higher recruitment scores for CD8 T cells, NK cells, T cells, and Th1 cells than those in the GLY^high^/TME^low^ subgroup.

### 3.6. High GLY Scores Were Associated with TME

**Figure 6:**
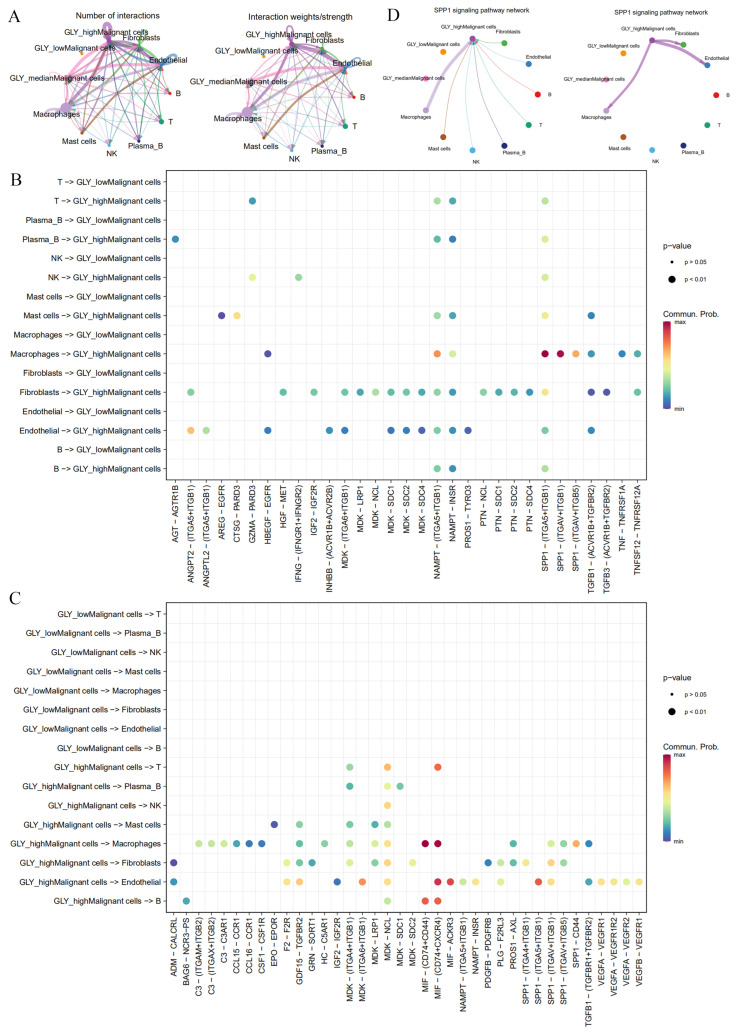
Differences in cell-to-cell communication between malignant cells with different GLY scores and cells within the TME. (**A**) Number and strength of cell-to-cell interactions. (**B**) LR pairs from immune cells and stromal cells to malignant cells. (**C**) LR pairs from malignant cells to immune cells and stromal cells. (**D**) SPP1 signaling pathways between GLY^high^ malignant cells and immune cells and stromal cells in HCC.

The next step was to determine the key players in the TME that contribute to glycosylation activity. Based on single-cell transcriptome data (GSE166635), we previously determined 10 cell subsets, including NK cells, plasma B cells, mast cells, macrophages, endothelial cells, B cells, fibroblasts, malignant cells, T cells, and epithelial cells. The GLY score is more highly expressed in malignant cell subsets than in other cells. Based on this, we divided malignant cell subsets into groups according to the GLY score: GLY^high^ malignant cells, GLY^median^ malignant cells, and GLY^low^ malignant cells. To characterize the cell-to-cell interactions of the three groups of GLY score malignant cells with other cell subsets, we used “CellChat” to infer possible cell-to-cell interaction signals as a function of ligand–receptor (LR) signaling. We detected boosted interactions between malignant and other cells in the high GLY score group, and the results showed that GLY^high^ malignant cells communicate extensively with fibroblasts, endothelial cells, and macrophages, followed by GLY^median^ malignant cells, whereas GLY^low^ malignant cells have no interaction information with other cells (Figure 6A). This finding indicates that GLY^high^ malignant cells have the potential to contribute to glycosylation-modified phenotypes.

We then examined the signal network of high and low GLY scores to identify individual LR pairs between malignant cells and other cells. Among them, fibroblasts, endothelial cells, and macrophages targeting GLY^high^ malignant cells work together through certain tumor-promoting signaling pathways, including the NAMPT-(ITGA5 + IAGB1), SPP1-(ITGA5 + ITGB1), NAMPT-INSR, and TGFB1 (ACVR1B + TGFBR2) signaling pairs, which interact to mediate uncontrolled tumor proliferation and growth (Figure 6B). The cell adhesion signaling pathway SPP1, the chemokine-related signaling pathways CCL15 and CCL16, the vascular endothelial growth factor VEGF signaling pathway, and the midkine (MDK) signaling pathway were the major signaling factors secreted by malignant cells with high GLY scores to other cell types (Figure 6C). Among them, the MDK signaling pathway has been reported as having a vital function in HCC occurrence and growth [30]. In addition, malignant cells with high GLY scores preferentially send signals to macrophages, endothelial cells, and fibroblasts through the SPP1 signaling pathway, while malignant cells with high GLY scores receive this signal mainly through macrophages (Figure 6D). In conclusion, GLY^high^ malignant cells may have more communication with immune cells and stromal cells in the TME than GLY^low^ malignant cells.

### 3.7. Therapy Prediction Based on the GLY/TME Classifier

**Figure 7:**
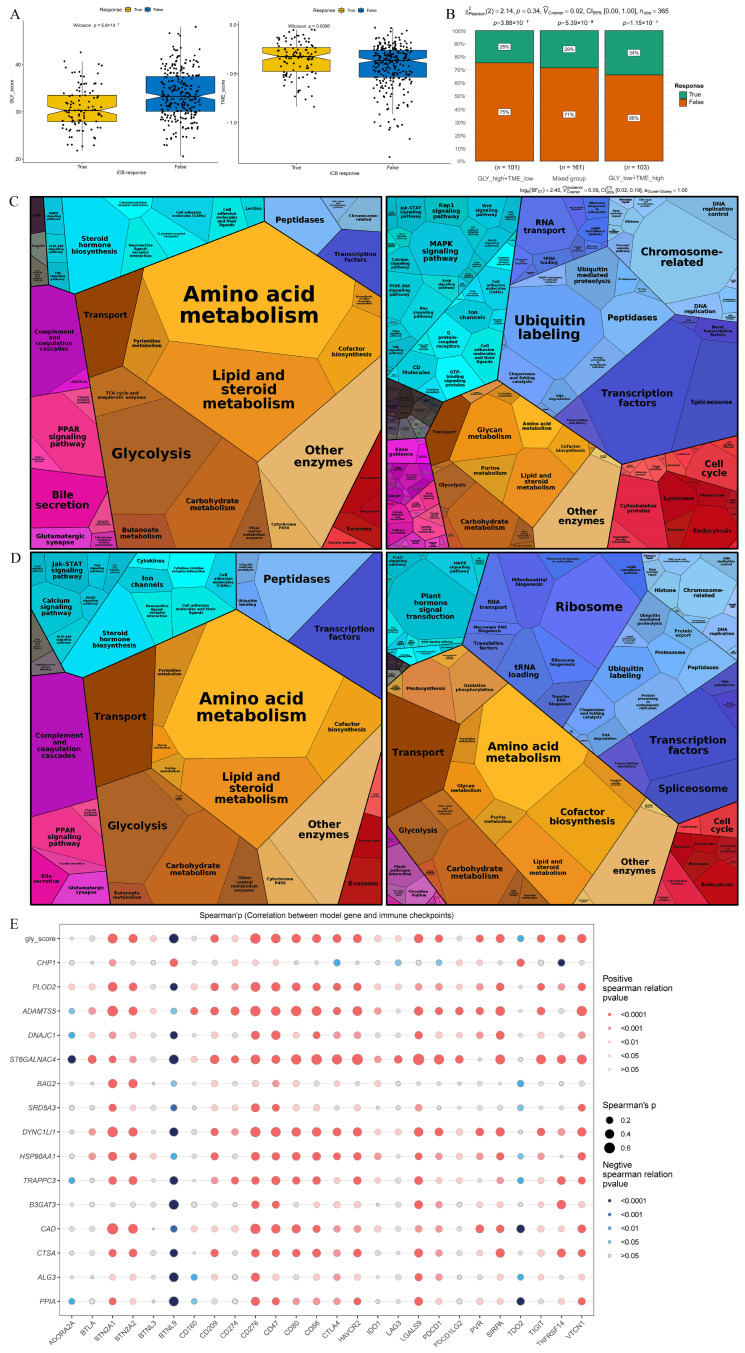
Prediction of treatment response based on the GLY/TME classifier. (**A**) The left panel represents the comparison of glycosylation-related gene scores. The right panel indicates the comparison of tumor microenvironment scores (true, responders; false, non-responders). (**B**) Different percentages of immunotherapy responders in subgroups based on the GLY/TME classifier (true, responders; false, non-responders). (**C**) Functional analysis of GLY^low^/TME^high^ patients receiving immunotherapy. The left is based on upregulated genes, and the right is based on downregulated genes. The size of each small polygon, representing a KEGG pathway, is correlated with the proportion of subgroups. (**D**) Functional analysis of patients responding to immunotherapy. The left is based on upregulated genes, and the right is based on downregulated genes. The size of each small polygon, representing a KEGG pathway, is correlated with the proportion of subgroups. (**E**) Correlation analysis between model gene and immune checkpoint.

TIDE analysis was employed to anticipate the response to immunotherapy of individuals with HCC in GLY score subgroups and TME score subgroups in order to investigate the function of the GLY/TME classifier in immunotherapy. According to the results, individuals with low GLY scores responded more favorably to immunotherapy than those with high GLY scores, suggesting that they may benefit more from immunotherapy. In addition, with respect to the TME score subgroups, individuals with high TME scores benefited from immunotherapy (Figure 7A). Moreover, 34% of GLY^low^/TME^high^ patients responded to immunotherapy, compared to 25% and 29% in the GLY^high^/TME^low^ and mixed groups, respectively, with a decreasing trend (Figure 7B). In addition, Proteomap was used to visually reveal the underlying mechanism by which the GLY/TME classifier predicts treatment response in patients receiving immunotherapy. By setting |logFC| > 0.5 and adj.pval < 0.05, we screened the differentially upregulated genes among the GLY^low^/TME^high^ and the GLY^high^/TME^low^ subgroups and the differentially upregulated genes between the immunotherapy response and non-response subgroups.

Common pathways were compared between the GLY^low^/TME^high^ subgroup and the immunotherapy response subgroup. Surprisingly, the Proteomap blocks of upregulated and downregulated genes were quite similar in the GLY^low^/TME^high^ (Figure 7C) and immunotherapy response groups (Figure 7D). In untreated HCC patients, the GLY/TME classifier features may be useful for predicting patient response to therapy. The two groups were also compared for immunological checkpoint expression, as immunological checkpoints are critical for effective immunotherapy. As shown in the bubble map, 26 genes involved in immune checkpoints were correlated with the model genes (Figure 7E). *PLOD2*, *ADAMTS5*, *ST6GALNAC4*, *DYNC1LI1*, *HSP90AA1*, and *CAD* were significantly correlated with immune checkpoint genes.

### 3.8. Benefit of Therapeutic Agents in GLYlow/TMEhigh and GLYhigh/TMElow Subgroups

**Figure 8:**
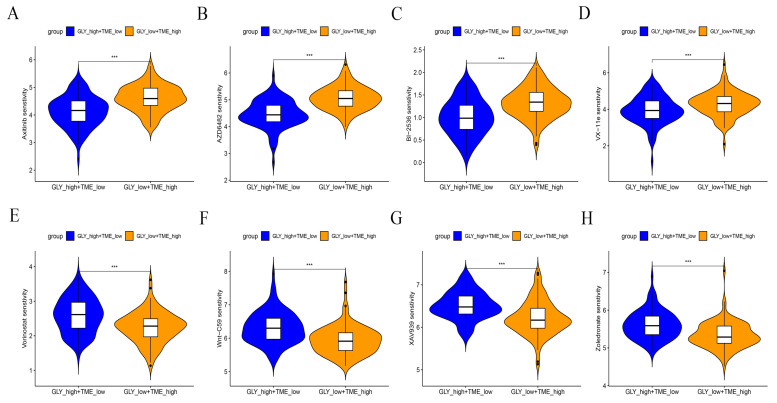
Drug sensitivity analysis of GLY^low^/TME^high^ patients versus GLY^high^/TME^low^ patients. (**A**–**H**) Response of the two subgroups to chemotherapeutic drugs. *p* values are shown with asterisks in the figure.

A drug sensitivity analysis was performed to analyze the sensitivity to chemotherapeutic agents of GLY^low^/TME^high^ patients in the GLY/TME score classifier versus GLY^high^/TME^low^ patients. The results showed that GLY^high^/TME^low^ patients were more sensitive to Axitinib, PI3K inhibitor (AZD6482), ERK inhibitor (VX-11e), and PLK inhibitor (BI-2536) and had better drug efficacy. In contrast, GLY^low^/TME^high^ patients were more sensitive to Zoledronate, Tankyrase inhibitor (XAV939), WNT inhibitor (WNT-C59), and Vorinostat (Figure 8A–H).

## 4. Discussion

Proteomics-based precision medicine is expanding quickly [31]. With the rapid growth of several omics, precision medicine strategies that take into account individual differences have become a promising approach to improve survival. Glycosylation is a vital component of protein posttranslational modification and plays important roles in metabolism, immune response, and malignancy [32,33,34,35,36]. A variety of enzymes, including glycosyltransferases and glycosidases, have a tight and dynamic function in glycosylation regulation [11]. However, most studies have focused on a single glycosyltransferase [37,38,39], and the overall metabolic and TME landscape characteristics mediated by signaling molecules in integrated glycosylation-related pathways are not fully understood. Therefore, gaining an understanding of the molecular mechanisms associated with glycosylation modifications from metabolic and tumor microenvironmental landscape features will help to improve our understanding of the function of glycosylation modifications in HCC and uncover new potential markers.

Aberrant glycosylation has been recognized as a novel cancer hallmark because of its crucial function in tumor biology [40]. At the same time, metabolic changes in tumor cells influence the activation, identification, proliferation, and cytotoxic capabilities of tumor-associated immune cells, helping them to evade immune surveillance [41,42]. Tumor cells alter the tumor microenvironment and help to create an immunosuppressive TME by restricting nutrients availability and by releasing metabolites, chemokines, and growth factors that reprogram the normal cell function and metabolism in TME [43].

Increased oxygen and glucose consumption by hyperproliferative tumor cells may deprive the TME of nutrients required by immune cells, reduce T cell cytotoxicity and effector activity, and cause the accumulation of immunosuppressive metabolites in the TME [44]. Shi et al. demonstrated that increased glucose uptake by M2-like tumor-associated macrophages (M2-like TAMs) promoted O-GlcNAcylation of cathepsin B via O-GlcNAc transferase (OGT), which increased mature cathepsin B levels in macrophages and its secretion into the TME, ultimately promoting cancer metastasis and chemoresistance [45]. Taken together, these findings suggest that metabolic reprogramming in cancer cells, including HCC, enhances the growth advantage of tumor cells and has significant effects on the TME and immune cells, promoting immunological escape [46,47,48]. Among these, the N-linked glycosylation alterations of PD-L1 enhance the evasion of T-cell-mediated immune responses and preserve its interaction with PD-1 [49].

Cascio et al. found that mucin 1 (MUC1) can bind to cell surface lectins such as CD169 through its surface modified glycosylation motifs. Furthermore, macrophages are significantly activated by the binding of CD169 to sialylated MUC1, thereby interfering with cancer immune surveillance and ultimately promoting tumor growth [50]. Nevertheless, few investigations have combined glycosylation modifications and TME signatures to predict HCC prognosis and therapeutic response. Here, we concentrate on the function of glycosylation-related genes and attempt to further evaluate the effects of glycosylation modification combined with the TME signature on the prognosis and immunotherapy response prediction of individuals with HCC by establishing a GLY/TME classifier using a large-scale HCC cohort.

Clinical efficacy and patient prognosis were better in the GLY^low^/TME^high^ subgroup. The prognostic capability of the classifier was further demonstrated in an independent cohort (GSE14520), demonstrating its broad applicability in cancer patients. This may indicate some common characteristics of host antitumor immune responses in glycosylation-related TMEs. Interestingly, among the 603 selected differentially expressed glycosylation-related genes, 12 genes included in the glycosylation-related scoring system were adverse prognostic factors and may be responsible for the metabolic reprogramming of tumorigenesis. Emerging research evidence has led to a growing recognition that aberrantly glycosylated proteins are regulators of malignant phenotypes in cancer cells [51]. Based on numerous studies showing that glycosylation modification contributes to the malignant phenotype of various cancer types, the 12 genes associated with glycosylation modification have been laterally identified as adverse prognostic factors.

Cancer-related signaling pathways and extracellular matrix (ECM)–receptor interaction mechanisms were substantially enriched in patients with high glycosylation scores, suggesting that enhanced glycosylation in the TME of HCC is beneficial for cancer progression. The N-glycosylation of integrin alpha 2 (ITGA2) affects cell–ECM adhesion, which promotes cancer metastasis by selectively adhering to ECM proteins [52]. Furthermore, our investigation showed a negative relationship between GLY score and TME score in the training cohort, which may indicate that abnormal glycosylation suppresses the antitumor immunological response. These outcomes all indicate the tumor biological value of the GLY/TME classifier. In addition, negative regulation of the cellular macromolecular biosynthesis process, mitotic G2 DNA damage checkpoint signaling, negative regulation of the mitotic cell cycle, and other biological processes connected to the inhibition of cell growth and proliferation progression were enriched in the GLY^low^/TME^high^ subgroup. This finding may elucidate the underlying mechanisms by which the GLY/TME classifier predicts prognosis and the immunotherapy effect.

HCC is a highly heterogeneous malignant tumor, and a more accurate classification system is needed to meet the needs of individualized treatment. Aiming at the tumor-specific targets of glycosylation modification, previous studies have focused on cancer diagnosis and prognostic stratification of comprehensive glycosylation regulatory signals and the application effect of models for immunotherapy, rarely considering their combination with glycosylation modification. A scoring system based on the immune infiltration characteristics of the TME has been established to stratify the prognosis of tumor patients. Zhou et al. identified a novel glycosyltransferase predictive signature as an independent poor predictor of OS in HCC [37]. Tang et al. established an HCC diagnostic model dependent on glycosylation regulation of relative expression orderings (REOs). The research team created a glycoscore system that can evaluate the various glycosylation characteristics of patients in the independent validation and discovery groups. The scoring system was also effective in predicting the treatment response to sorafenib, transarterial chemoembolization (TACE), and anti-PD-1 therapy with different glycosylation patterns [53]. Lv et al. established a model according to glycosyltransferase characteristics to predict breast cancer prognosis and response to immunological checkpoint inhibitors and verified that the high-risk group in comparison was associated with low OS, weak immune effect, high chemical sensitivity, and different CNV mutation patterns [38]. Sha et al. showed that glycosyltransferase (GT)-based GT score was associated with GT expression and neuroblastoma (NB) prognosis, disialoganglioside phenotype, and immune infiltration, providing new evidence for predicting NB prognosis and response to immunotherapy [39]. By using in silico approaches, Caputo et al. identified hub genes and transcription factors including *AURKA*, *CCNB1*, *CDK1*, *RRM2*, and *TOP2A* as promising candidates for potential drug testing [54]. Huang et al. found that *POLE2*, *GABARAPL1*, *PIK3R1*, *NDC80*, and *TPX2* play critical roles as potential biomarkers to enhance the immunotherapeutic role of PD-L1 inhibitors [55]. These novel therapeutic approaches have the potential to be tested in a specific cohort of patients with HCC following successful experimental validation.

In contrast, our study established a glycosylation-related score by integrating signaling molecules in glycosylation–related pathways and established a TME immune infiltration score by screening immune-infiltrating cells related to a good prognosis. Three major categories were employed to stratify HCC patients’ prognosis. Among them, the GLY^low^/TME^high^ subgroup had the longest survival, showing excellent prognostic prediction performance. Regarding immune infiltration, the GLY^low^/TME^high^ subgroup had elevated recruiting values for CD8 T, NK, T, and Th1 cells, indicating that this group of patients had good antitumor immunity, which was consistent with the expected effect, suggesting the feasibility of the model to anticipate the prognosis of HCC patients.

We further analyzed the main cell types enriched by the glycosylation score at the single-cell level, and the results showed that the enrichment of tumor malignant epithelial cells was the highest. The subgroup was divided into three cell types according to the GLY score: GLY^high^ malignant cells, GLY^median^ malignant cells, and GLY^low^ malignant cells. Cell communication analysis was conducted to further discover the interaction between this subgroup and other cell groups in the TME. Interestingly, the communication between GLY^high^ malignant cells and fibroblasts, endothelial cells, and macrophages was significantly enhanced, followed by GLY^median^ malignant cells, whereas GLY^low^ malignant cells had no interaction information with other cells. Combined with the score enrichment degree of each cell subset above, these results indicate that GLY^high^ malignant cells are a significant target of glycosylation modification.

Xu et al. found that PLOD2 expression was positively correlated with 15 immune checkpoint genes in patients with stomach adenocarcinoma (STAD) [56]. For the first time, based on analysis, we found that PLOD2 expression was positively correlated with 12 immune checkpoint genes (*BTN2A1*, *BTN2A2*, *CD209*, *CD276*, *CD47*, *CD80*, *CD86*, *CTLA4*, *HAVCR2*, *SIRPA*, *TIGIT*, *VYCN1*) in patients with HCC.

CHP1 has been identified as an important regulator of endoplasmic reticulum (ER) glycerolipid synthesis and assists in the complete glycosylation of Na^+^/H^+^ exchanger isoform 1 (NHE1) [57,58]. In recent study, Xi et al. employed transcriptome expression data from the hepatocellular carcinoma cancer genome map (TCGA-LIHC) to develop a model consisting of 5 NK cell-related genes (*IL18RAP*, *CHP1*, *VAMP2*, *PIC3R1*, *PRKCD*), which divided patients into high- and low-risk groups based on their risk score [59].

Although our study showed encouraging predictive results, there are still some limitations. First, we used bioinformatics methods to explore the heterogeneity risk predictive ability of glycosylation modification and TME infiltration in HCC. Future collection of a larger sample size is needed to improve the prediction efficiency and accuracy. Second, more laboratory tests are needed to validate the precise biological processes of the discovered genes. In conclusion, the analysis of glycosylation changes and immune cell infiltration in the TME may help to predict prognosis and response to therapy, which may be a promising tool for prognostic evaluation and patient stratification in future medical treatment of the disease.

## 5. Conclusions

In conclusion, our results suggest that the model constructed with glycosylation (GLY) and the tumor microenvironment (TME) can well predict the prognosis of HCC patients.

## Data Availability

The data presented in this study are available on request from the corresponding author. The data are not publicly available due to better management of data sharing and to support the better understanding of raw data.

## Supplementary Materials

The following supporting information can be downloaded at https://www.mdpi.com/article/10.3390/metabo14010051/s1, Figure S1: The differential expression of these glycosylation-related genes in HCC compared to normal paracancerous tissues; Figure S2: The prognostic and predictive significance of the GLY/TME classifier; Table S1: Glycosylation-related pathway genes; Table S2: RT-PCR primer; Table S3: Differentially expressed glycosylation-related genes associated with prognosis title; Table S4: Detail information of 15 genes in the risk signature.References [60,61,62,63,64,65,66,67,68,69,70,71,72,73] are cited in the Supplementary Materials.

## Abbreviations

HCC, hepatocellular carcinoma; TILs, tumor-infiltrating lymphocytes; GLY, Glycosylation; TME, tumor microenvironment; TIME, tumor immune microenvironment; LIHC, Liver Hepatocellular Carcinoma; TCGA, The Cancer Genome Atlas; ROC, receiver operating characteristic; TPM, transcripts per million; MsigDB, Molecular Signatures Database; UCSC, University of California, Santa Cruz; t-SNE, t-distributed stochastic neighbor embedding; KM, Kaplan–Meier; NK cells, natural killer cells; DEGs, differentially expressed genes; LASSO, least absolute shrinkage and selection operator; FGSEA, fast gene set enrichment analysis; KEGG, Kyoto Encyclopedia of Genes and Genomes; CPTAC, Clinical Proteomic Tumor Analysis Consortium; OGT, O-GlcNAc transferase; NICD, Notch intracellular domain; DFS, disease-free survival; TAMs, tumor-associated macrophages; MHC, major histocompatibility complex; ITGA2, integrin alpha 2; REOs, relative expression orderings; TACE, transarterial chemoembolization; GTs, glycosyltransferases; NB, neuroblastoma; CIBERSORT, Cell-type Identification By Estimating Relative Subsets Of RNA Transcripts; OS, overall survival; WGCNA, Weighted Gene Coexpression Network Analysis; TOM, topological overlap measure; GSEA, gene set enrichment analysis; TIP, tumor immunophenotype; TIDE, Tumor Immune Dysfunction and Exclusion; GDSC, Genomics of Drug Sensitivity in Cancer Project; ECM, extracellular matrix; ITGA2, integrin alpha 2; REOs, relative expression orderings; TACE, transarterial chemoembolization; NHE1, Na+/H+ exchanger isoform 1; STAD, stomach adenocarcinoma.
