# Supplementary material for: Integrating TCGA and Single-Cell Sequencing Data for Hepatocellular Carcinoma: A Novel Glycosylation (GLY)/Tumor Microenvironment (TME) Classifier to Predict Prognosis and Immunotherapy Response
Source: Metabolites. 2024 Jan 13;14(1):51. doi: 10.3390/metabo14010051 (PMC10818448; doi:10.3390/metabo14010051)
Supplement: Supplementary file 1 [file metabolites-14-00051-s001.zip › Supplementary Materials.pdf]

Supplementary Materials

Integrating TCGA and Single-Cell Sequencing Data for Hepatocellular Carcinoma: A Novel Glycosylation (GLY)/Tumor Microenvironment (TME) Classifier to Predict Prognosis and Immunotherapy Response

Yun Wu [1,†], Jiaru Chen [1,2,†], Riting Zhu [1,2], Guoliang Huang [1], Jincheng Zeng [1,3], Hongbing Yu [1], Zhiwei He [1,*] and Cuifang Han [1,*]

[1] Guangdong Provincial Key Laboratory of Medical Molecular Diagnostics, Guangdong Medical University, Dongguan 523808, China; wuyun@gdmu.edu.cn (Y.W.); cjrkkl@gdmu.edu.cn (J.C.); zhuriting1998@gdmu.edu.cn (R.Z.); huangguoliang@gdmu.edu.cn (G.H.); zengjc@gdmu.edu.cn (J.Z.); hbyu@gdmu.edu.cn (H.Y.)
[2] School of Pharmacy, Guangdong Medical University, Dongguan 523808, China
[3] Dongguan Key Laboratory of Medical Bioactive Molecular Developmental and Translational Research, Guangdong Medical University, Dongguan 523808, China
* Correspondence: hezhiwei@gdmu.edu.cn (Z.H.); hancuifang@gdmu.edu.cn (C.H.)
† These authors contributed equally to this work.

1 Supplementary Figures and Tables

1.1 Supplementary Tables

Supplementary Table S1. Glycosylation-Related Pathway Genes. The TCGA database contains clinical information of various cancer types that is kept by UCSC Xena, and we obtained the genomic information and medical annotations of liver hepatocellular carcinoma (LIHC) individuals, which included LIHC (n = 374) and normal (n = 50) samples, and then performed log2 TPM (transcripts per kilobase million) +1 formats for each expression value. In total, 770 glycosylation-related genes were collected from the Molecular Signatures Database (MSigDB).

Supplementary Table S2. RT-PCR primer. mRNA level of the target and normalization to that of beta-actin mRNA level were detected by quantitative real-time PCR (qRT-PCR) using the 2−ΔΔCt method and the primers are listed in Table S2.

Supplementary Table S3. Differentially expressed glycosylation-related genes associated with prognosis. 301 DEGs associated with prognosis were screened out by univariate Cox analysis, and the differential expression of these glycosylation-related genes in HCC compared to normal paracancerous tissues was shown using a heatmap.

Supplementary Table S4. Detail information of 15 genes in the risk signature. A robust prognostic gene signature was constructed with 15 genes, namely, PPIA, ALG3, CTSA, CAD, B3GAT3, TRAPPC3, HSP90AA1, SRD5A3, BAG2, DNAJC1, ADAMTS5, PLOD2, DYNC1LI1, ST6GALNAC4, and CHP1, and detailed information about them is provided in Table S4.

1.2 Supplementary Figures

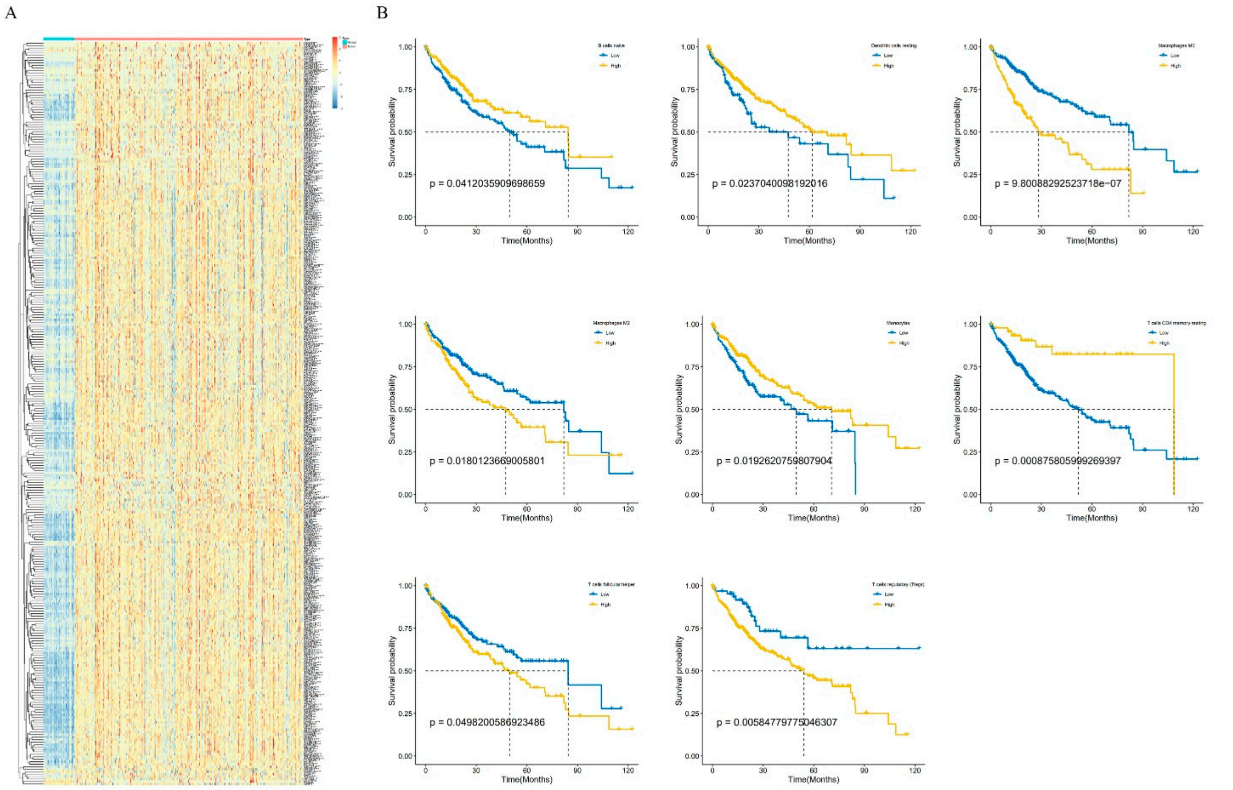

Supplementary Figure S1. The differential expression of these glycosylation-related genes in HCC compared to normal paracancerous tissues. (A) Heatmap showing the expression of 301 differentially expressed glycosylation-related pathway genes in HCC tumor samples compared with normal samples. (B) Kaplan–Meier analysis of overall survival according to the level of infiltration by immune cells in HCC, including naive B cells, resting DCs, M0 macrophages, M2 macrophages, monocytes, resting memory

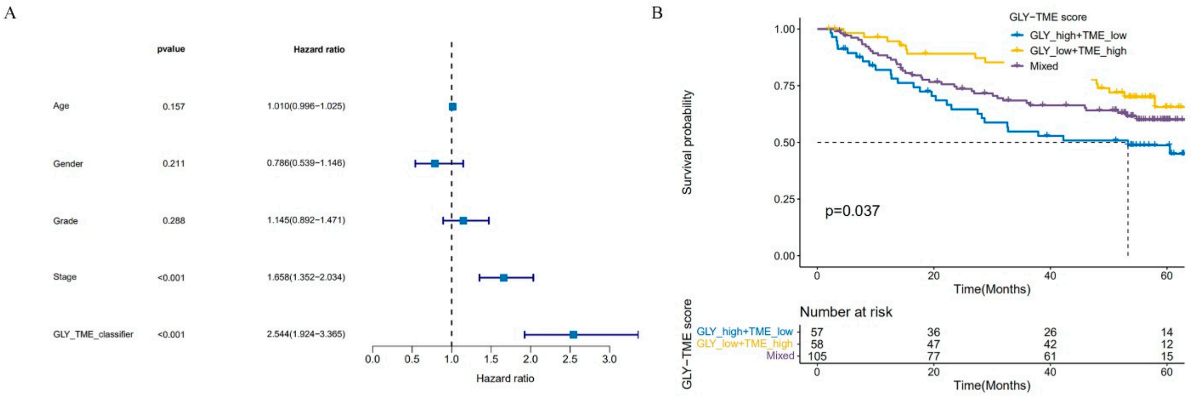

Supplementary Figure S2. The prognostic and predictive significance of the GLY/TME classifier. (A) Univariate Cox regression analysis of the GLY-TME classifier in the training cohort. (B) Kaplan-Meier survival curves of the validation cohort were stratified into three different subgroups (GLY^{low}/TME^{high}, mixed and GLY^{high}/TME^{low}) according to the GLY-TME classifier. Log-rank test, $p < 0.001$.
