# Supplementary material for: Integrating TCGA and Single-Cell Sequencing Data for Hepatocellular Carcinoma: A Novel Glycosylation (GLY)/Tumor Microenvironment (TME) Classifier to Predict Prognosis and Immunotherapy Response
Source: Metabolites. 2024 Jan 13;14(1):51. doi: 10.3390/metabo14010051 (PMC10818448; doi:10.3390/metabo14010051)
Supplement: Supplementary file 1 [file metabolites-14-00051-s001.zip › Supplementary Table S2 RT-PCR primer.docx]

Table S2. Primer sequences of HCC prognostic genes and internal reference gene beta Actin (ACTB)

| Primer name | Sense | Anti-sense |
| --- | --- | --- |
| PPIA | GGCAAATGCTGGACCCAACACA | TGCTGGTCTTGCCATTCCTGGA |
| ALG3 | CTTTGCTGTGCTCTACCTGGCT | CGCAGCACAAAGATGGAGTGGA |
| CTSA | GCTTCGTGAAGGAGTTCTCCCA | CTGTGGTCATCAGTATGGCTGC |
| CAD | TAGTCCTTGGCTCTGGCGTCTA | TAGTCGGTGCTGACTGTCTCTG |
| B3GAT3 | GCCCTTGCTGTTAGATAAGCCC | GCTTCTCTGTCCGAGTATGCCA |
| TRAPPC3 | CTGGTCACCCAGCTATGTAAGG | ACCTCCCAACATTTGACCGAGC |
| HSP90AA1 | TCTGCCTCTGGTGATGAGATGG | CGTTCCACAAAGGCTGAGTTAGC |
| SRD5A3 | TCTTCCAGGACCTGATCCGCTA | CAGGAAGCCATTCCACAGCACT |
| BAG2 | TAGCCAGGACATGAGGCAGATC | GCTTTAGGGATTCTTGCTGCTGG |
| DNAJC1 | GGACTCCAGGTCGATGGGAAAA | GGAGCAGGTCACTGAATCCTTC |
| ADAMTS5 | CCTGGTCCAAATGCACTTCAGC | TCGTAGGTCTGTCCTGGGAGTT |
| PLOD2 | GACAGCGTTCTCTTCGTCCTCA | CTCCAGCCTTTTCGTGGTGACT |
| DYNC1LI1 | GCAAAGCAACCACCAACTGCAG | ATGGGTGACACGCTGGCAACAT |
| TUBULIN | TTGGGAGGTCATCAGCGATGAG | AGGCTCCAGATCCACCAGGATG |
| ST6GALNAC4 | ACACCTTCACGGAGCGCATGAT | CCATAGACCACGATCTCCTCAC |
| ACTB | CACCATTGGCAATGAGCGGTTC | AGGTCTTTGCGGATGTCCACGT |
