# Supplementary material for: Integrating TCGA and Single-Cell Sequencing Data for Hepatocellular Carcinoma: A Novel Glycosylation (GLY)/Tumor Microenvironment (TME) Classifier to Predict Prognosis and Immunotherapy Response
Source: Metabolites. 2024 Jan 13;14(1):51. doi: 10.3390/metabo14010051 (PMC10818448; doi:10.3390/metabo14010051)
Supplement: Supplementary file 1 [file metabolites-14-00051-s001.zip › Supplementary Table S4 Detail information of 15 genes in the risk signature.docx]

Supplementary Table S4 | Detail information of 15 genes in the risk signature.

| Gene symbol | Full name | Function of the encoded protein |
| --- | --- | --- |
| *PPIA* | Peptidylprolyl isomerase A | CypA is incorporated into the HIV type 1 (HIV-1) virion and promotes HIV-1 infectivity by facilitating virus uncoating.(1) |
| *ALG3* | Alpha‐1,3‐mannosyltransferase | ALG3 catalyzes the first Dol-P-Man-dependent mannosylation step, which is critical for lipid-linked oligosaccharides and protein N-linked glycosylation, at the luminal side of the ER.(2) |
| *CTSA* | Cathepsin A | CTSA is a serine protease cathepsin member of the cathepsin lysosomal protease family, which with a role in protecting β-galactosidase and neuraminidase-1 from intra-lysosomal proteolysis.(3) |
| *CAD* | Carbamoyl-phosphate synthetase 2, aspartate transcarbamylase and dihydroorotase | CAD is a single large polypeptide with three highly conserved enzymatic activities: carbamoyl phosphate synthase (CPS2), aspartate transcarbamylase (ATCase), Dihydroorotase (DHOase); these are the first three enzymes of de novo pyrimidine, and the name of CAD is the abbreviation of these enzymes.(4) |
| *B3GAT3* | Beta-1,3-glucuronyltransferase 3 | GlcAT-I completes the last step of transfer of a glucuronic acid (GlcA) from the donor substrate uridine diphosphate-glucuronic acid (UDP-GlcUA) to the linkage region Gal-β-(1-3)-Gal-β-(1-4)-Xyl.(5) |
| *TRAPPC3* | Trafficking protein particle complex subunit 3 | TRAPPC3 is important for membrane tethering events in the formation of the pre-Golgi compartment or vesicular tubular clusters (VTCs).(6) |
| *HSP90AA1* | Heat shock protein 90 alpha family class A member 1 | HSP90 is a group of inducible ATP‐dependent molecular chaperones in response to cellular stress. Four isoforms of HSP90 have been identified in mammalian cells, including Hsp90α (encoded by HSP90AA1).(7) |
| *SRD5A3* | Steroid 5 alpha-reductase 3 | SRD5A3 codes for polyprenol reductase which converts polyprenol to dolichol. This is a major pathway for dolichol biosynthesis for N-glycosylation, O-mannosylation, C-mannosylation, and GPI anchor synthesis.(8) |
| *BAG2* | Bcl-2 Associated Athanogene 2 | BAG2, as an anti-apoptotic gene, can promote cell proliferation, inhibit cell apoptosis and arrest the cell cycle, and a raised BAG2 expression is found in a number of tumor types, such as thyroid cancer and breast cancer.(9) |
| *DNAJC1* | DnaJ heat shock protein family (Hsp40) member C1 | DNAJC1 encodes a member of the heat shock family proteins (hsp), which are well characterized in stress and immune responses, and its transcripts and proteins are highly expressed in thyroid samples.(10) |
| *ADAMTS5* | A disintegrin and metalloproteinase with thrombospondin motifs 5 | ADAMTSs are a family of zinc metalloendopeptidases that participate in diverse biological processes, such as procollagen processing, ECM remodeling, inflammation, cell migration, and vascular biological processes. In particular, ADAMTS5 overexpression is a key risk factor in degenerative joint diseases and intervertebral disc degeneration.(11) |
| *PLOD2* | Procollagen-Lysine,2-Oxoglutarate 5-Dioxygenase 2 | The accumulation of stabilized collagen is enhanced by different covalent collagen cross-links, lysyl hydroxylases 2 (encoded by the PLOD2 gene) is the key enzyme mediating the formation of the stabilized collagen cross-link. Interestingly, PLOD2 is overexpressed in different cancers and closely related to a poor prognosis.(12) |
| *DYNC1LI1* | Dynein cytoplasmic 1 light intermediate chain 1 | Dync1li1, a subunit of cytoplasmic dynein 1, is reported to play important roles in intracellular retrograde transport in many tissues.(13) |
| *ST6GALNAC4* | ST6 (alpha-N-acetyl-neuraminyl-2,3-beta-galactosyl-1,3)-N-acetylgalactosaminide alpha-2,6-sialyltransferase 4 | ST6GALNAC4 catalyzes the addition of sialic acid (Neu5Ac) to the 69 carbon of N-acetylgalactosamine (GalNAc) of the acceptor motif Neu5Ac-alpha-2,3-Gal-beta-1,3-GalNAc on glycoproteins and glycolipids, resulting in a terminal, diallylated glycan structures.(14) |
| *CHP1* | Calcineurin B homologous protein 1 | CHP1 binds and activates GPAT4, which catalyzes the initial rate-limiting step in glycerolipid synthesis.(15) |

# References

1. An P, Wang LH, Hutcheson-Dilks H, Nelson G, Donfield S, Goedert JJ, et al. Regulatory polymorphisms in the cyclophilin A gene, PPIA, accelerate progression to AIDS. *PLoS Pathog* (2007) 3(6):e88. doi: 10.1371/journal.ppat.0030088.

2. Liu P, Lin C, Liu Z, Zhu C, Lin Z, Xu D, et al. Inhibition of ALG3 stimulates cancer cell immunogenic ferroptosis to potentiate immunotherapy. *Cell Mol Life Sci* (2022) 79(7):352. doi: 10.1007/s00018-022-04365-4.

3. Wang D, Zaitsev S, Taylor G, D'azzo A, Bonten E. Protective protein/cathepsin A rescues N-glycosylation defects in neuraminidase-1. *Biochim Biophys Acta* (2009) 1790(4):275-82. doi: 10.1016/j.bbagen.2009.01.006.

4. Shin J, Mir H, Khurram MA, Fujihara KM, Dynlacht BD, Cardozo TJ, et al. Allosteric regulation of CAD modulates de novo pyrimidine synthesis during the cell cycle. *Nat Metab* (2023). doi: 10.1038/s42255-023-00735-9.

5. Haltiwanger RS, Lowe JB. Role of glycosylation in development. *Annu Rev Biochem* (2004) 73:491-537. doi: 10.1146/annurev.biochem.73.011303.074043.

6. Barrowman J, Bhandari D, Reinisch K, Ferro-Novick S. TRAPP complexes in membrane traffic: convergence through a common Rab. *Nat Rev Mol Cell Biol* (2010) 11(11):759-63. doi: 10.1038/nrm2999.

7. Song Q, Wen J, Li W, Xue J, Zhang Y, Liu H, et al. HSP90 promotes radioresistance of cervical cancer cells via reducing FBXO6-mediated CD147 polyubiquitination. *Cancer Sci* (2022) 113(4):1463-74. doi: 10.1111/cas.15269.

8. Wheeler PG, Ng BG, Sanford L, Sutton VR, Bartholomew DW, Pastore MT, et al. SRD5A3-CDG: Expanding the phenotype of a congenital disorder of glycosylation with emphasis on adult onset features. *Am J Med Genet A* (2016) 170(12):3165-71. doi: 10.1002/ajmg.a.37875.

9. Pattingre S, Turtoi A. BAG Family Members as Mitophagy Regulators in Mammals. *Cells* (2022) 11(4). doi: 10.3390/cells11040681.

10. Huang L, Yu Z, Zhang T, Zhao X, Huang G. HSP40 interacts with pyruvate kinase M2 and regulates glycolysis and cell proliferation in tumor cells. *PLoS One* (2014) 9(3):e92949. doi: 10.1371/journal.pone.0092949.

11. Clement-Lacroix P, Little CB, Smith MM, Cottereaux C, Merciris D, Meurisse S, et al. Pharmacological characterization of GLPG1972/S201086, a potent and selective small-molecule inhibitor of ADAMTS5. *Osteoarthritis Cartilage* (2022) 30(2):291-301. doi: 10.1016/j.joca.2021.08.012.

12. Du H, Pang M, Hou X, Yuan S, Sun L. PLOD2 in cancer research. *Biomed Pharmacother* (2017) 90:670-6. doi: 10.1016/j.biopha.2017.04.023.

13. Zhang Y, Zhang S, Zhou H, Ma X, Wu L, Tian M, et al. Dync1li1 is required for the survival of mammalian cochlear hair cells by regulating the transportation of autophagosomes. *PLoS Genet* (2022) 18(6):e1010232. doi: 10.1371/journal.pgen.1010232.

14. Chang LY, Liang SY, Lu SC, Tseng HC, Tsai HY, Tang CJ, et al. Molecular Basis and Role of Siglec-7 Ligand Expression on Chronic Lymphocytic Leukemia B Cells. *Front Immunol* (2022) 13:840388. doi: 10.3389/fimmu.2022.840388.

15. Zhu XG, Nicholson Puthenveedu S, Shen Y, La K, Ozlu C, Wang T, et al. CHP1 Regulates Compartmentalized Glycerolipid Synthesis by Activating GPAT4. *Mol Cell* (2019) 74(1):45-58 e7. doi: 10.1016/j.molcel.2019.01.037.
